# Microvascular Anastomosis of Calcified Vessels in a Patient with Hyperlipidemia: Meeting the Challenges and Dealing with **Y** -Shaped Vein Graft

**DOI:** 10.1055/s-0044-1801790

**Published:** 2025-02-24

**Authors:** Raj Kumar Manas, Karthick Ganesan

**Affiliations:** 1 Department of Plastic, Reconstructive & Burns Surgery, All India Institute of Medical Sciences, New Delhi, India

**Keywords:** **Y** -shaped vein graft, atherosclerotic calcified vessel microanastomosis, hyperlipidemia and free flap

## Abstract

Hyperlipidemia is one of the causes of atherosclerotic calcified vessels. Anastomosis of such vessels during surgery may offer various challenges. It is twice as challenging when the limb's and flap's vascularity is compromised together. We present a case of microvascular anastomosis of calcified vessels with compromised limb vascularity (due to an inadvertent injury to the brachial artery, which created a gap between two ends) and low ischemia time for flap (free functioning muscle transfer) that dealt with a **Y** -shaped vein graft. Using a **Y** -shaped vein graft in atherosclerotic vessels allowed us to anastomosis with ease and deal with challenges encountered during anastomosis. The **Y** -shaped vein graft was used to reconstruct the brachial artery and at the same time, another limb of the **Y** -shaped vein graft was used to anastomose the flap's vessel. Postoperatively vascularity of the limb and flap were restored and computed tomography angiography confirmed patent anastomosis of the **Y** -shaped vein graft. The **Y** -shaped vein graft is a novel and simple technique that can be utilized when dealing with calcified vessel anastomosis and can help in restoring the blood supply of the flap and limb together.

## Introduction

Microvascular anastomosis of the calcified vessels is a difficult task for microsurgeons involved in reconstructive microsurgery. It becomes more challenging when there is less ischemia time for flaps like free functioning muscle transfer (FFMT) and at the same time, there is a concern for limb viability. The presence of atheromatous plaque in a patient with hyperlipidemia makes the recipient vessels stiff, difficult to mobilize, difficult to handle, and difficult to suture, and there is more risk of thrombosis.

We present a case of micro-arterial anastomosis of severely calcified vessels using a **Y** -shaped interposition vein graft for flap and for restoring the blood supply to the upper limb. The aim of reporting this case is to highlight the utility of the **Y** -shaped vein graft where the two distal limbs of Y were utilized to restore the blood supply of two different tissues, one of which was the free-functioning muscle, and at the same time restore the compromised blood supply of the limb due to an advertent injury to the brachial artery.

## Case Report

**Fig. 1:**
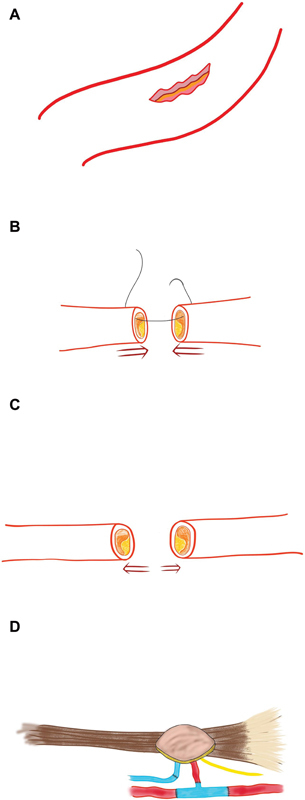
( **A** ) Schematic diagram showing a tear in the brachial artery with the presence of plaque inside. ( **B** ) An attempt to repair the two arterial ends primarily after the excision of plaque at the injured site. ( **C** ) The two cut ends of the artery retract back due to stiffness and difficulty in mobilizing them. ( **D** ) Use of **Y** -shaped vein graft ( *blue colored* ) interposed between two cut ends of the artery ( *red colored* ) and another limb of the vein connected with the artery of Gracilis flap.

A 28-year-old man of average build with a history of brachial plexus injury was posted for elective FFMT. There was no history of diabetes, peripheral vascular disease, or significant smoking. However, the patient had incidental findings of multiple lipomas in the body. The peripheral pulses were palpable clinically, so computed tomography (CT) angiography was not done. The pulsation and flow were checked and were found suitable from the recipient vessel, which was the proximal branch of the brachial artery (namely, the profunda brachii artery in the arm with an average diameter of 2.5 mm) before flap harvest and anastomosis. However, following anastomosis with the recipient artery end to end, there was no flow at the anastomosis site. So, a few millimeters of adventitia was stripped of the proximal branch of the brachial artery with the hope of relieving vasospasm and enhancing the flow. During the handling of the vessels, there was a small iatrogenic puncture in the brachial artery (just below the junction where the proximal branch was emerging), which continued to leak (challenge 1), and the subsequent attempt to repair the hole causing the leak turned into a big laceration (challenge 2). Despite multiple attempts, the tear was difficult to repair and a plaque was seen in the lumen with a separated layer of intima ( Fig. 1A ; challenge 3). Another attempt was made to excise the plaque area and repair the artery primarily ( Fig. 1B ; challenge 4). But that could not be done as the two ends of the brachial artery retracted back and were difficult to mobilize ( Fig. 1C ) due to stiffness, thus creating a gap of 4 cm (challenge 5). The limb turned pale with no capillary refill, and the ischemia time for flap was approaching, which is less in FFMT (45 minutes to 1 hour).

## Innovations



**Video 1** This video demonstrates the reconstruction of the brachial artery using a Y-shaped vein graft, with the second distal limb of the vein graft anastomosed to the flap's artery.


**Video 2** The Acland test shows a patent anastomosis, with confirmation of flap vascularity indicated by a bright red bleed from the flap's margin.


**Fig. 2:**
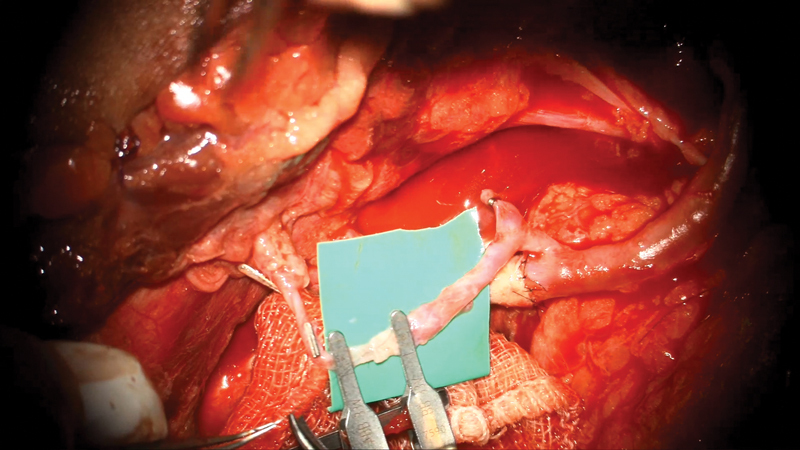
Vein graft connected with the distal end of the brachial artery. Another limb of the **Y** -shaped vein graft connected to the flap vessel (arterial clamp in situ).

A decision to maintain the continuity of the brachial artery was made using a saphenous vein graft measuring 4 cm (after reversing the ends) and another *drain-out branch* (which was confirmed by filling with heparinized saline into the main vein graft and the branch that became dilated served as a drain-out branch) of the saphenous vein graft forming a Y (∼2 cm), which was used to anastomose the flap's vessel ( Figs. 1D and 2 ; Video 1 ). The sutures used were Nylon 9–0 with a tapered cutting end, as available in our setup. Following anastomosis, the capillary refill was restored and the flap bled a bright red color from the margin intraoperatively ( Video 2 ). The total ischemia time of restoring arterial inflow was 1 hour.

The venous drainage flap was maintained with a vein connecting the subcutaneous vein in the arm. Postoperatively, the limb and the flap perfused well. The flap and the limb were monitored every 1 to 2 hours for the first 2 days and then every 4 hours for the next 3 days.

**Fig. 3:**
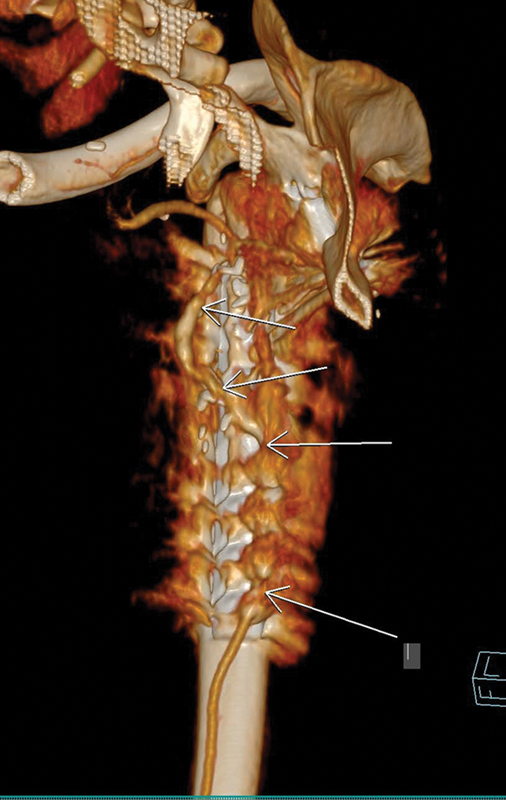
3D volume rendering reformatted image showing patency of vein graft and flap's vessel.

**Fig. 4:**
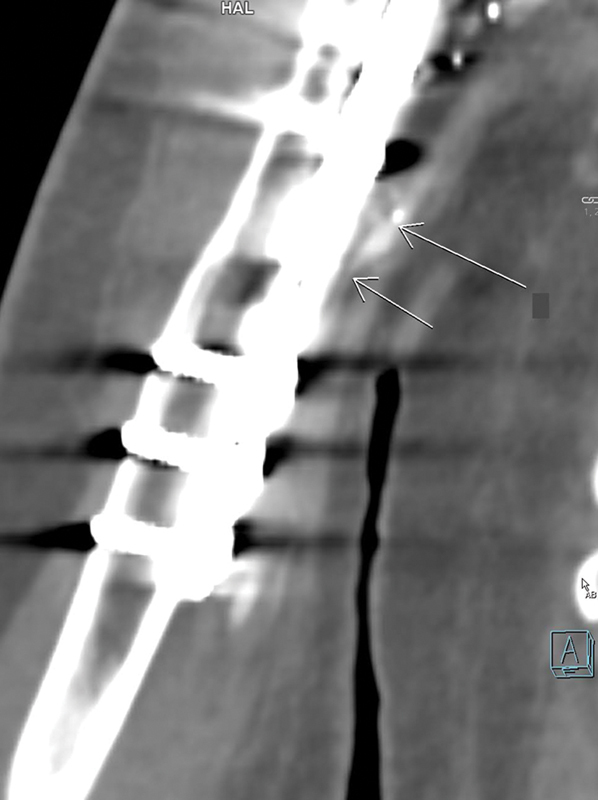
Computed tomography angiography showing patency of the vein graft along with patency of the flap's vessel.

Retrospectively, we analyzed all possible causes of the atheromatous plaque formation. Due to multiple lipomas, we investigated for lipid profile, which was found deranged, and a diagnosis of hyperlipidemia was made. Postoperatively, CT angiography confirmed the patency of the brachial artery and the flap artery using a vein graft ( Figs. 3 and 4 ).

## Discussion

Atheromatous plaque formation in the calcified vessels may be due to old age, diabetes mellitus, peripheral vascular disease, smoking habit, and hyperlipidemia, which may affect microvascular anastomosis during free flap surgery. Hyperlipidemia as a risk factor for free flap complications has been noted in various studies. 1 However, calcified vessels are not contraindications to microsurgery, but most of the time, when it is encountered intraoperatively, it may present with various challenges. A quick decision based on proposed guidelines and experiences can help in facing such challenges and overriding the problems that may happen during surgery. Also, in case of clinical suspicion in history that suggests the formation of plaque and calcification of vessels, one should confirm it preoperatively with color Doppler or CT angiography to see the luminal patency or atheroma formation.

Umezawa and Ogawa shared tips for microsurgical arterial anastomosis in severe atherosclerosis patients and advised avoiding anastomosis at calcified lesions. However, when required, the smallest and lightest but adequate vascular clamps are to be applied. Also, the needle should pass from the adventitia through to the inner intimal lining of vessels, with adequate forces according to the curvature of the needle. 2 One should also avoid adventitial stripping during dissection and can palpate the vessels for thickening of the wall. However, we could not appreciate the atherosclerotic wall during dissection and were carried away with adventitial stripping that led to a series of challenges we encountered.

Bouaoud et al also shared the same key points for managing calcifications in head and neck cases. Apart from avoiding arterial dilatations using lidocaine, and papaverine (as there is no vasospasm in calcified vessels), they recommend avoiding clamps. In the end, fibrin sealant may help ensure the pedicle position. 3 Cai et al described a case report of suturing the intima with media with interrupting sutures and thus preventing curling of the intima and prolapsing of the plaque inside the lumen, which this facilitates anastomosis. 4

Since such an artery becomes stiff, it becomes difficult to mobilize; the use of a vein graft allows the recipient vessels to anastomose more freely. Periasamy et al described the scout vein graft used to assess the suitability of atherosclerotic donor arteries where 4 to 5 cm of vein graft is connected at the donor artery in an end-to-side fashion and the flow is assessed before proceeding for flap harvest. If a donor vessel is found suitable, which is confirmed through a vein graft, anastomosis of the flap vessels is done with a vein graft that provides at least one pliable surface, which makes the anastomosis easier and patent. 5

Hosein et al described venous interposition grafting as a novel technique for microsurgery in calcified vessels. The venous interposition graft provides better ergonomics to facilitate tensionless anastomosis across arterial gaps and less damage to the intimal endothelium. They also found that vein grafts improve the seal at the anastomosis site to avoid leakage compared to rigid calcified arteries. 6

Our technique of using interposition graft was similar to Tsao et al's description of using a **Y** -shaped vein graft in drain-out branches of the vein graft as additional sources for free flap reconstruction in injured lower extremities. 7 The difference with our case was that the vascularity of the limb was preserved in their cases due to the presence of other arteries and was used for lower limb defects. On the other hand, in our case, the vascularity was required to be restored due to injury of a single supply by the brachial artery and at the same the ischemia time of the transferred FFMT (gracilis muscle) was approaching. Utilizing a **Y** -shaped vein graft helped us to face such challenges and restore the blood supply of the limb and flap successfully.

## Conclusion

Utilizing a vein graft can be a novel technique when dealing with arteriosclerotic calcified vessels. It allows more compliance during anastomosis, with less risk of separation of fragile intima, and deals with the challenge(s) encountered during calcified vessel anastomosis.
